# Stealthing among university students: associated factors

**DOI:** 10.1590/1980-220X-REEUSP-2021-0573en

**Published:** 2022-06-10

**Authors:** Gleicy Kelly Felix Costa, Monalisa Nanaina da Silva, Ana Paula Rodrigues Arciprete, Juliana Cristina dos Santos Monteiro

**Affiliations:** 1 Universidade de São Paulo, Enfermagem pela Escola de Enfermagem de Ribeirão Preto, Ribeirão Preto, SP, Brazil.; 2 Universidade de São Paulo, Escola de Enfermagem de Ribeirão Preto, Programa de Pós-Graduação Enfermagem em Saúde Pública, Ribeirão Preto, SP, Brazil.; 3 Universidade de São Paulo, Escola de Enfermagem de Ribeirão Preto, Departamento de Enfermagem Materno-Infantil e Saúde Pública, Ribeirão Preto, SP, Brazil.

**Keywords:** Students, University, Sexual and Reproductive Health, Gender-Based Violence, Condoms, Sex Offences, Estudiantes, Universidades, Salud Sexual y Reproductiva, Violencia de Género, Condones, Delitos Sexuales, Estudantes, Universidades, Saúde Sexual e Reprodutiva, Violência de Gênero, Preservativos, Delitos Sexuais

## Abstract

**Objective:**

To identify the practice of stealthing among university students and the associations between the profile of these young people and this practice.

**Method:**

Cross-sectional study carried out at a university campus in a city in the countryside of Sao Paulo. Data collection was carried out online by RedCap between May and September 2018, through questionnaires with identification data, sociodemographic characteristics and sexual and reproductive health. Data were analyzed by IBM-SPSS, version 17.0.

**Results:**

A total of 380 students participated in the study, aged between 18 and 24 years old, most of them unpaid students, coming from private education, not having a religion and being single. Most of them were biologically female and identified as cisgender and heterosexual women. As for stealthing, 1.33% of the participants had performed it and 11.44% had already undergone this practice. There was a significant association between having been stealthed and the variables female biological sex (p = 0.000) and identifying as a woman (p = 0.000).

**Conclusion:**

The occurrence of stealthing is higher among those who have been stealthed than among those who have done it and having been stealthed is associated with being female and identifying as a woman.

## RESUMO

**Objetivo:**

Identificar a prática de *stealthing* entre jovens universitários e as associações entre o perfil desses jovens e a prática do *stealthing*.

**Método:**

Estudo transversal realizado em um campus universitário de um município no interior paulista. A coleta de dados foi *online* pelo RedCap, entre maio e setembro de 2018, por meio de questionários com dados de identificação, características sociodemográficas e de saúde sexual e reprodutiva. Os dados foram analisados pelo IBM-SPSS, versão 17.0.

**Resultados:**

Participaram do estudo 380 estudantes, com idade entre 18 e 24 anos, a maioria sem exercer função remunerada, advindos(as) de ensino particular, sem religião e solteiras(os). Em sua maioria, eram do sexo biológico feminino e se identificavam como mulheres cisgênero e heterossexuais. Quanto ao *stealthing*, 1,33% dos participantes tinham realizado e 11,44% já tinham sofrido essa prática. Houve associação significativa entre ter sofrido *stealthing* e as variáveis sexo biológico feminino (p = 0,000) e se identificar como mulher (p = 0,000).

**Conclusão:**

A ocorrência do *stealthing* é maior entre os que sofreram essa prática do que entre aqueles que a praticaram. Ter sofrido *stealthing* está associado a ser do sexo feminino e se identificar como mulher.

## RESUMEN

**Objetivo:**

Identificar el *stealthing* entre jóvenes universitarios y la asociación entre el perfil de esos jóvenes y su práctica.

**Método:**

Se trata de un estudio transversal realizado en un campus universitario de un municipio del interior de São Paulo. Los datos se recopilaron en línea, en el RedCap, entre mayo y septiembre de 2018 mediante cuestionarios con datos de identificación, características sociodemográficas, salud sexual y reproductiva, y se analizaron con el IBM-SPSS, versión 17.0.

**Resultados:**

Participaron del estudio 380 estudiantes, con edades comprendidas entre 18 y 24 años, gran parte sin trabajo remunerado, provenientes de la enseñanza particular, sin religión y solteras(os). En su mayoría eran del sexo femenino biológico y se identificaban como mujeres cisgénero y heterosexuales. En cuanto al *stealthing*, el 1,33% de los participantes lo había realizado y el 11,44% ya había sufrido esta práctica. Hubo una asociación significativa entre haber sufrido *stealthing* y las variables sexo biológico femenino (p = 0,000) y el identificarse como mujer (p = 0,000).

**Conclusión:**

La incidencia del *stealthing* es mayor entre los que han sufrido esta práctica que entre los que la han practicado. Haber sufrido el furtivismo se asocia a ser mujer y a identificarse como tal.

## INTRODUCTION

The transition from adolescence to youth is constituted by physical changes such as the rapid growth and maturation of secondary sexual characteristics and also by the construction of personality, adaptation to the environment and the search for social interaction for greater acceptance of behaviors considered as expected for an adult^(1)^. However, even though the development of sexuality is something natural and expected, there is an early involvement of young people in sexual relations and with the implication of risks and health problems^(1)^.

With regard to the experiences of sexuality among adolescents and young people, it is evident that among many who start their sexual life there is a lack of information about counseling services, which increases their vulnerability in relation to Sexually Transmitted Infections (STIs) and to unwanted pregnancies^(2)^. Thus, promoting sexual and reproductive health is to offer conditions for these young people to have a safe sexual initiation, through actions of inclusion, information, education and health care, in the context of life in which this young person is inserted, allowing them to do choices safely, so as not to compromise their health in the exercise of their sexuality^(2)^.

For young people who enter university, this phase is marked by significant changes in their lifestyle, their autonomy and changes in parental limits, which can influence their behavior in a way that has implications for health^(3)^. In a study on risk behaviors related to the health of university students, alcohol consumption appeared as the most prevalent risk behavior^(3)^. High alcohol consumption predisposes to the aggravation of other problems such as unprotected sexual behavior, which is a prevalent behavior among adolescents and young people^(4)^.

The process that involves sexual initiation, with experimentation and learning, consists of factors such as reproduction and contraception, influenced and determined by gender issues that may be associated with vulnerabilities and violence, among other markers^(5)^ like ethnicity/race and generation. In addition to health risk behavior issues, women are even more vulnerable due to biological and social factors^(6)^ that lead to gender inequalities and are directly reflected in the sexual and reproductive health of these young women^(7)^.

The concept of gender is a constitutive element of social relationships, based on the differences that are perceived between the genders^(8)^. Through gender analysis, it is possible to understand, visualize and refer to the social organization of the relationship between the sexes^(9)^.

It is important to reflect and consider in the social environment, that the gender identity reflected by biological sex only fits the “female” and the “male”, hindering the social and political inclusion of people who do not fit into this binary structure^(10)^. In addition, this limited classification socially defines how bodies should flow in relation to sexuality and aspects of human reproduction^(9)^ and it is a challenge in the political, ethical and scientific fields to demystify the rooted and disseminated stereotypes of a certain hierarchical biological order that standardizes bodies, sex and spaces for human reproduction, imposing social orders and norms on these bodies that provide different, but limited, social experiences^(5)^. Reproductive health policies, aimed at the young population, cannot refrain from debating fundamental issues such as gender, sexuality and gender violence, including among university students^(10)^.

At the center of this debate, among other issues, consent is essential. Socially, it is up to women to consent or not to sexual practices, which also involves issues of gender hierarchies, interwoven by sociosexual determinations that go far beyond wanting or simply accepting to have sexual relations^(11)^. It should be noted that “accepting” involves an alienation of will, desire and the ultimate goal of the sexual relationship, which would be, in addition to the reproductive issue, a source of pleasure^(11)^.

However, considering the historically constructed and ingrained misconduct, for a long time there was no conception of consent or not on the part of women in the face of men’s intentions to obtain sexual satisfaction, even through constraint or physical force, which is still reproduced today in some love, affective or sexual relationships^(12)^. Thus, as gender violence is characterized by acts of psychological, physical, moral and sexual violence against women, in the domestic, family context or in intimate relationships of affection, their rights are often implicitly or explicitly violated. However, there are still many weaknesses in the elaboration and implementation of public policies that are effective in dealing with gender violence^(13)^.

Consent goes beyond saying yes or no to the sexual act, and within relationships, a risky practice called stealthing emerges, which is the removal of a condom during sexual intercourse without the partner’s consent. Thus, a consensual sexual relationship can become a non-consensual sexual relationship, being recurrent among young people, in relationships in which most of the time the victims do not perceive the partner’s behavior and when they perceive the practice and deny the continuity of the sexual act, the victim suffers even more violence from the partner or strong psychological threat to continue the sexual act^(14)^.

The term emerged in the United States of America, where legislators are trying to categorize stealthing as a form of sexual assault, due to the non-consensual nature of this act and the physical and moral damages it can cause to the victim. However, although in the American context there is a consensus that the practice falls within the scope of gender violence against women, there is a difficulty in considering it a crime when it is understood by some as a misconduct, and not primarily as a violation of rights of women^(15)^. Yet, without a standardized translation in Brazil and not effectively recognized as a crime, the practice of stealthing is common among young people, exposing women to the risk of unwanted pregnancy and women and men to STIs, being a violation of trust and denial of autonomy, as well as rape^(16)^. However, stealthing is still little discussed and studied nationally and internationally as a crime and sexual violence^(14,15)^ and for this reason this study proposes to carry out a survey of the practice of stealthing among young university students, from a gender perspective, analyzing factors that may be related to this risky practice.

Therefore, this study aims to identify the practice of stealthing among university students and the associations between the profile of these young people and the practice of stealthing.

## METHOD

### Design of Study

This is an observational, cross-sectional, descriptive and analytical study.

### Local

The study was carried out on the campus of a public state university, in a big city in the countryside of the state of Sao Paulo, where 28 on-site undergraduate courses are offered and there is a distance learning center.

### Population and Sample

The study population consisted of all undergraduate students from the university campus enrolled in the first semester of 2018, which had a total of 6969 undergraduate students. Students aged between 18 and 24 years old were invited to participate, and those who reported being under 18 or 25 years old or older, and who had not started their sexual life at the time of data collection, were excluded. A total of 1025 entries were obtained in the search. Considering the completed questionnaires and following the inclusion and exclusion criteria, the sample of this study had 380 participants.

### Data Collection

For data collection, a structured questionnaire was used with identification data, sociodemographic characteristics and sexual and reproductive health characteristics of the participants, prepared based on scientific literature and existing previous research. The sociodemographic characteristics studied were: age, color or self-reported race, course and year of course, religion, occupation, smoking habit, drinking habit, habit of using illicit drugs, marital status, type of school previously attended (public or private) and family income. The sexual and reproductive health characteristics studied were: biological sex, sexual orientation, gender identity (gender with which the individual identifies, regardless of biological sex), age at which sexual intercourse began, number of sexual partners at lifetime, use of contraceptive methods and its type, reason for not using a contraceptive method, use or not of contraceptive methods by the partner, use or not of condoms (internal or external), reason for not using condoms, use by the partner, occurrence of sex under the influence of alcohol or other illicit drugs, occurrence of sex in which the partner was under the influence of alcohol or other illicit drugs, desire to have sexual intercourse and sensation of sexual intercourse and the practice of stealthing (performed or suffered).

The questionnaire was transferred to the Research Electronic Data Capture (REDCap) tool. Agility during collection, better data quality, greater control of the research process and time savings in obtaining results faster are some of the benefits of this tool^(17)^.

Data collection took place between May and September 2018, when students were invited to participate via email, receiving all information about the study and an email address that directed them to the online survey page. After being aware of the research objectives and ethical aspects, those who agreed to participate expressed it electronically, by clicking on the acceptance button on the page containing the Informed Consent Form (ICF). After that, there were the online pages that contained the research instruments, self-completed instruments, in which the participant read and answered, without the intervention of the researcher.

### Data Analysis and Treatment

Data were imported directly from REDCap to a structured electronic spreadsheet in Microsoft Excel and analyzed using the statistical program Statistical Package for Social Sciences, SPSS, version 17.0. To characterize the sample and to identify the other variables studied, the analysis was based on descriptive statistics, through univariate data analysis. Fisher’s exact test was used to analyze the relationship between the variables. For all analyses, a significance level of 5% was considered.

### Ethical Aspects

Regarding ethical aspects, the project was conceived and executed according to the ethical requirements recommended by Resolution No. 466 of 2012, of the National Health Council, having been submitted and approved by the Research Ethics Committee of the Ribeirao Preto College of Nursing from the University of Sao Paulo, linked to the National Research Ethics Commission (CONEP), under opinion N. 4331209 issued on October 9, 2020.

As already described, on the online survey page, after the participants were aware of the ethical aspects, the participants clicked on the acceptance of participation button on the page containing the TCLE and only after reading and confirming their agreement, they proceed to the search form pages.

## RESULTS

The mean age of the participants was 21 years old with a standard deviation of 2.03 years old. Table 1 shows that most participants self-declared as white (78.16%), did not have paid work (80.00%), had no religion (79.21%), and declared themselves single (51.58%). Regarding studies, the highest percentage (41.58%) had studied fully in private schools.

**Table 1.** Distribution of participants according to self-reported color, work, religion, marital status and type of school they attended – Ribeirao Preto, SP, Brazil, 2018.

Self-reported color (n = 380)	(N = 308)
White	297 (78.16%)
Black	15 (3.95%)
Brown	55 (14.47%)
Yellow	13 (3.42%)
**Do you carry out paid work?**	
Yes	76 (20.00%)
No	304 (80.00%)
**Do you actively participate in any religion?**	
Yes	79 (20.79%)
No	301 (79.21%)
**Marital Status**	
Single without partner	196 (51.58%)
I have a partner, but we don’t live together	168 (44.21%)
Married or living with a partner	16 (4.21%)
**Regarding your years of study, which one is your group? (n = 380)**	
I studied fully in a private school	158 (41.58%)
I studied most of the time in a private school	71 (18.68%)
I studied fully in public school	108 (28.42%)
I studied most of the time in public school	43 (11.32%)

Regarding the characteristics of lifestyle habits, most participants reported not having the habit of smoking (88.42%) or using illicit drugs (66.94%). Regarding alcoholic beverages, most of them reported consumption, and the highest percentage of participants (36.84%) reported using these beverages once or twice a week. Table 2 presents the distribution of study participants regarding these variables.

**Table 2.** Distribution of participants according to smoking, alcohol consumption and illicit drug use – Ribeirao Preto, SP, Brazil, 2018.

Smoking habit	(N = 308)
Yes	44 (11.58%)
No	336 (88.42%)
**Alcohol consumption habit**	
Drinking every or almost every day	5 (1.32%)
Drinking once or twice a week	140 (36.84%)
Drinking once or twice a month	119 (31.32%)
Drinking less than once a month	57 (15.00%)
Do not drink	59 (15.53%)
**Use of illicit drugs**	
Using every or almost every day	12 (3.16%)
Using once or twice/week	15 (3.95%)
Using one to three times/month	31 (8.16%)
Using less than once/month	68 (17.89%)
Do not use	254 (66.94%)

Regarding sexual and reproductive health characteristics, we emphasize that, in this study, all participants had already started their sexual life. Regarding biological sex, 253 (66.58%) young people were female and 127 (33.42%) were male. Regarding gender identity, 255 (67.11%) identified themselves as cisgender women and 125 (32.89%) identified themselves as cisgender men, that is, two participants declared themselves as transgender women. The mean age at first sexual intercourse was 17 years old, with a standard deviation of 1.92, median of 17.00 years old, minimum of 9 and maximum of 24 years old. Regarding the number of partners during life, the average was 7, with a standard deviation of 12.84, with the minimum of one partner and the maximum of 150 (which was reported by only one person).

Table 3 presents the distribution of participants according to sexual orientation, use of contraceptive methods (except condoms), use of condoms, choice to use condoms, sensations during sexual intercourse and desire to have sexual intercourse.

**Table 3.** Distribution of participants according to sexual orientation, use of contraceptive methods (except condoms), use of condoms, choice to use condoms, sensations during sexual intercourse and desire to have sexual intercourse – Ribeirao Preto, SP, Brazil, 2018.

Sexual orientation (N = 380)	N(%)
Heterosexual	284 (74.74%)
Homosexual	27 (7.11%)
Bisexual	67 (17.63%)
Asexual	2 (0.53)
**Use of contraceptive methods (N = 380)**	
Yes	310 (81.58%)
No	70 (18.42%)
**Use of condom (N = 378)**	
Always	145 (38.36%)
Most of the time	124 (32.80%)
Sometimes	65 (17.20%)
No	44 (11.64%)
**Option to use condoms (N = 334)**	
Mine	64 (19.16%)
My partner’s	6 (1.80%)
Both	264 (79.04%)
**Sensations during intercourse (N = 374)**	
Pleasure	333 (89.04%)
Pain	15 (4.01%)
None	20 (5.35%)
Others	6 (1.60%)
**Desire to have sexual intercourse (N = 374)**	
Always	117 (31.28%)
Most of the time	163 (43.58%)
Sometimes	89 (23.80%)
Never	5 (1.34%)

Most participants reported being heterosexual (74.74%) and using some contraceptive method (81.58%), with 38.36% always saying they used condoms in their sexual relations, 79.04% shared the decision for the use of condoms with partner. With regard to sensations during sexual intercourse, most participants (89.04%) reported feeling pleasure and as for the desire to have sexual intercourse, 43.58% reported having it most of the time.

Regarding practices during sexual intercourse, we investigated among university students whether they had ever performed or suffered stealthing and whether they had ever had sex under the influence of alcohol or other drugs, as shown in Table 4.

**Table 4.** Distribution of study participants in terms of variables having performed and having undergone stealthing and engaging in sexual intercourse under the influence of alcohol or other drugs – Ribeirao Preto, SP, Brazil, 2018.

Have you ever removed a condom during sexual intercourse without your partner’s consent?? (N = 377)	N(%)
Yes	5 (1.33%)
No	335 (88.86%)
Does not apply	37 (9.81%)
**Has a partner ever removed a condom during sexual intercourse without your consent? (N = 376)**	
Yes	43 (11.44%)
No	316 (84.04%)
Does not apply	17 (4.52%)
**Have you ever had sex under the influence of alcohol or other drugs? (N = 377)**	
Yes	260 (68.97%)
No	117 (31.03%)
**Have you ever had sex with a partner who was under the influence of alcohol or other drugs? (N = 376)**	
Yes	250 (66.49%)
No	126 (33.51%)

Regarding the performance of stealthing, it was identified that 1.33% of the sample had performed this practice and 11.44% had already suffered this situation. Regarding the practice of sexual intercourses under the influence of alcohol or other drugs, 68.97% had already gone through it.

An association was made between the practice of stealthing (performed or suffered) and the selected sociodemographic and lifestyle variables. According to Table 5, there was a statistically significant result for the association of the variable having been stealthed with the variables biological sex (p = 0.000) and gender identity (p = 0.000). That is, having suffered stealthing was associated with being women and identifying as a woman.

**Table 5.** Association between the practice of stealthing (performed or suffered) with the selected sociodemographic and lifestyle variables – Ribeirao Preto, SP, Brazil, 2018.

	Already performed stealthing (N = 340)		p*	Already suffered stealthing (N = 359)		p*
	Yes	No		Yes	No	
**Self-reported color**						
White and Yellow	3 (0.88%)	275 (80.88%)	0.226	34 (9.47%)	260 (72.42%)	0.672
Black and Brown	2 (0.59%)	60 (17.65%)		9 (2.51%)	56 (15.60%)	
**Religion**						
Has religion	2 (0.59%)	64 (18.82%)	0.250	6 (1.67%)	62 (17.27%)	0.533
Has no religion	3 (0.88%)	271 (79.71%)		37(10.31%)	254 (70.75%)	
**Biological sex**						
Female	4 (1.18%)	213 (62.65%)	0.657	40 (11.14%)	200 (55.71%)	0.000
Male	1 (0.29%)	122 (35.88%)		3 (0.84%)	116 (32.31%)	
**Gender identity**						
Woman	4 (1.18%)	215 (63.24%)	0,658	40 (11.14%)	202 (56.27%)	0.000
Man	1 (0.29%)	120 (35.29%)		3 (0.84%)	114 (31.75%)	
**Marital status**						
No partner	3 (0.88%)	177 (52.06%)	1	24 (6.69%)	165 (45.96%)	0.745
With a partner	2 (0.59%)	158 (46.47%)		19 (5.29%)	151 (42.06%)	
**Smoking habit**						
Yes	0 (0.00%)	41 (12.06%)	1	4 (1.11%)	39 (10.86%)	0.802
No	5 (1.47%)	294 (86.47%)		39 (10.86%)	277 (77.16%)	
**Habit of consuming alcoholic beverages**						
Yes	5 (1.47%)	282 (82.94%)	1	39 (10.86%)	266 (74.09%)	0.363
No	0 (0.00%)	53 (15.59%)		4 (1.11%)	50 (13.93%)	
**Use of illicit drugs**						
Yes	1 (0.29%)	112 (32.94%)	1	12 (3.34%)	110 (30.64%)	0.397
No	4 (1.18%)	223 (65.59%)		31 (8.64%)	206 (57.38%)	
**Have you ever had sex under the influence of alcohol or other drugs?**						
Yes	4 (1.18%)	234 (69.03%)	1	33 (9.19%)	219 (61.00%)	0.376
No	1 (0.29%)	100 (29.50%)		10 (2.79%)	97 (27.02%)	
**Have you ever had sex with a partner who was under the influence of alcohol or other drugs?**						
Yes	4 (1.18%)	224 (66.27%)	1	33 (9.22%)	209 (58.38%)	0.223
No	1 (0.30%)	109 (32.25%)		10 (2.79%)	106 (29.61%)	

* Fisher’s Exact Test.

## DISCUSSION

The study included a total of 380 university students, most of them were white, did not have paid work, had no religion, were single or without a partner and studied most of their lives in private schools. Thus, the findings of this study regarding these characteristics of the profile of university students are in accordance with the fact that, although the number of black students within higher education institutions in Brazil is increasing, most of them are white^(18)^. Despite not being the focus of this study, the profile of the participants refers to a reflection on the inequities present in society, emphasizing the intersectionality of gender, ethnicity/race and class, which maintain the hegemony of a white, CIS heteronormative and elite.

Regarding the consumption of alcoholic beverages, although 15.53% reported not using it, this data draws attention, since in Brazil alcohol consumption by university students is higher when compared to the general population^(19)^. With regard to the use of illicit drugs, 3.16% said they use them every or almost every day, a percentage that even calls attention to the possibility of these young people, under the influence of these drugs, putting themselves in risk situations, such as unprotected sexual intercourse^(20)^.

With regard to sexual and reproductive health characteristics, most of the students (74.74%) declared themselves to be heterosexual, unlike another study on sexual and reproductive health among university students, which identified 97.1% of heterosexual participants^(21)^.

Regarding the use of contraceptive methods other than condoms, 81.58% of the students reported using them. However, knowledge about contraceptive methods, as well as the right way to use them, do not necessarily make the contraceptive practice effective^(22)^. Regarding the use of external or internal condoms, 38.36% of the participants declared that they always use it, followed by 32.80% that they use it sometimes, and among those who use it, 79.04% reported that it is an option of both partners. Regarding the negotiation of condom use, some authors point out that among men there is a belief in the need to use condoms only in relationships with women considered by them as unreliable^(23)^. Trust, in this case, is associated with knowing and maintaining a stable and exclusive affective and sexual relationship with a person. On the other hand, the lack of trust is related to sporadic sexual relations and that can expose them to risk situations such as contamination by STIs and unwanted pregnancy^(23)^.

As for sensations during sexual intercourse, 89.04% reported feeling pleasure and 31.28% always reported feeling the urge to have sexual intercourse, followed by 43.58% who felt the urge most of the time. In a study carried out with Mexican university students, 78.5% claimed to have excellent or good quality of sex life^(24)^, corroborating this study, despite having used different research methods.

Regarding stealthing, 1.33% answered that they had already practiced it. On the other hand, 11.44% reported having already suffered, corroborating US findings, where 12.2% of young women had already suffered, and this percentage is lower than in Australia, where the practice suffered was reported by 32% of participants^(16)^. The situation of being stealthed demonstrates that, while consent is usually a non-verbal phenomenon, women often do not feel that they have the power to direct sexual intercourse. The act of consenting is considered a woman’s responsibility, and it is often up to men to negotiate this consent, as a way of exercising their power, which is built within the dynamics of domination and hierarchy of genders, but is often mistakenly considered to be as something associated with the biological, something inherent in the characteristics of sexual differentiation, which allows them to perform practices if consent is not explicit, leading to the naturalization of practices that are no less violent or risky^(11)^

In this study, stealthing was associated with female biological sex (p = 0.000) and female gender identity (p = 0.000), with no other statistically significant associations for the other selected variables. Gender-based violence can be difficult to prove, as is the case with stealthing. From this perspective, the practice of stealthing is not usually seen as a violation of women’s fundamental rights due to the heteropatriarchal construction, which validates and legitimizes this behavior, but which can generate serious social and health problems^(25)^.

As a limitation of this study, we can point out the lack of control over open forms, but not filled in, which restricted the number of participants included in the study. In addition, the income variable had to be disregarded, as the way in which the question was prepared allowed the insertion of random values, which were not possible to be analyzed.

## CONCLUSION

It is concluded that, among university students from the studied campus, the occurrence of stealthing is higher among those who suffered the practice than among those who practiced it. Furthermore, being stealthed is associated with being female and identifying as female.

It is evident that stealthing needs to be discussed more in our society and that there are educational actions in sexual and reproductive health formulated, considering gender and sexual and reproductive justice issues in order to allow young people to understand the risks and nature of sex violence linked to this practice. The empowerment and instrumentalization of women so that they can understand what consent involves and how to face this type of violence is an urgent issue so that social mechanisms can evolve to the point of allowing women and men to have sex in a safe and satisfying way.

## ASSOCIATE EDITOR

Rosa Maria Godoy Serpa da Fonseca
